# The burden of unintended pregnancies among Indian adolescent girls in Bihar and Uttar Pradesh: findings from the UDAYA survey (2015–16 & 2018–19)

**DOI:** 10.1186/s13690-023-01077-4

**Published:** 2023-04-27

**Authors:** Himani Sharma, Shri Kant Singh

**Affiliations:** Department of Survey Research and Data Analytics, International Institute for Population Sciences, Mumbai, India

**Keywords:** Unintended, Pregnancy, Young, Adolescents, India, UDAYA

## Abstract

**Background:**

Unintended pregnancy severely affects the health and welfare of women and children, specifically if women are young and vulnerable. This study aims to determine the prevalence of unintended pregnancy and its determinants among adolescent girls and young adult females in Bihar and Uttar Pradesh. We believe the present study is unique as it examines the association between unintended pregnancy and sociodemographic factors among young female population in two states of India from 2015–19.

**Methods:**

The data for the present study is derived from the two-wave longitudinal survey “Understanding the lives of adolescents and young adults” (UDAYA) conducted in 2015–16 (Wave 1) and 2018–19 (Wave 2). Univariate, bivariate analysis along with logistic regression models were employed.

**Results:**

The results revealed that 40.1 per cent of all currently pregnant adolescents and young adult females reported their pregnancy as unintended (mistimed and unwanted) in Uttar Pradesh at Wave 1 of the survey, which decreased to 34.2 per cent at Wave 2. On the contrary, almost 99 per cent of all currently pregnant adolescents in Bihar reported their pregnancy as unintended at Wave 1, which decreased to 44.8 per cent at Wave 2. The sociodemographic factors like age, caste, religion, education, wealth, media and internet use, knowledge and effective contraception highly impacted unintended pregnancy in Bihar and Uttar Pradesh. The longitudinal results of the study revealed that place of residence, internet use, number of wanted children, heard about contraception and SATHIYA, use of contraception, side effects of contraception, and the confidence in getting contraceptives from ASHA/ANM did not appear significant predictors at Wave 1. However, they emerge significant over time (Wave 2).

**Conclusions:**

Despite many recently launched policies for adolescents and the youth population, this study comprehended that the level of unintended pregnancies in Bihar and Uttar Pradesh stands worrisome. Therefore, adolescents and young females need more comprehensive family planning services to improve their awareness and knowledge about contraceptive methods and use.

## Plain English summary

Unintended pregnancy among adolescents represents a significant public health challenge in high-income and middle- and low‐income countries. Many kinds of research have been conducted in Africa, Latin America and other developing countries, keeping in mind the importance of sexual and reproductive behavior and the rights of adolescents and youth in their countries.

There are a few studies in India on unintended pregnancies, but none of them have special concerns about the adolescent and young female population. The current study, therefore, aims to study the prevalence of unintended pregnancy among adolescent girls and young females, examine the association between sociodemographic variables and unintended pregnancy and analyze the important predictors of these pregnancies among adolescent girls and young females in Uttar Pradesh and Bihar.

The data used in the present study is taken from the Population Council’s longitudinal survey called Understanding the lives of adolescents and young adults (UDAYA) conducted in 2015-16 and 2018-19. In order to determine the factors affecting unintended pregnancies among adolescents in Bihar and Uttar Pradesh, socioeconomic, demographic, health and contraception-related variables were taken into the analysis. Univariate, bivariate analysis along with binary logistic regression has been employed in the study. The results revealed that a significant proportion of pregnant women in Uttar Pradesh and Bihar report their current pregnancy as unintended at both Wave 1 and 2. It further added that age, caste, religion, education, wealth, media and internet use, knowledge and effective contraception broadly impact unintended pregnancy in the two socially and economically less empowered states of Bihar and Uttar Pradesh.

## Introduction

The instances of unintended pregnancy are shared by women worldwide, irrespective of their geographical location, per capita income and other demographics. Unintended pregnancy among adolescents represents a significant public health challenge in high-income and middle- and low‐income countries [1]. Adolescents constitute a large and important part of India’s population (approximately 253 million),and every fifth person in India is between 10 to 19 years [2]. Approximately 121 million unintended pregnancies occurred annually between 2015 and 2019 in low and middle-income countries across the globe [3]. The most recent report by the Guttmacher Institute reveals that the global unintended pregnancy rate has declined from 1990–1994 from 79 to 64 per 1,000 in 2015–19 in women of reproductive age (15–49) [3]. Despite this decline in unintended pregnancies, the proportion of unintended pregnancies remains high in developing countries. Nearly 40 per cent of the pregnancies in developing countries are unintended- either not wanted or mistimed [4]. According to a study conducted in India, 70 unintended pregnancies per 1000 women aged 15–49 years in India, translating to almost half of India's 48.1 million pregnancies in 2015, were unintended [5]. These unintended pregnancies lead to 25 million unsafe abortions and about 47,000 maternal deaths yearly [6]. Not just national-level surveys, the state-level surveys also portray the same picture. A state-level survey conducted in 2015 in the six Indian states entitled 'Unintended Pregnancy and Abortion in India' (UPAI) presents the estimates of abortion and unintended pregnancies in facility and non-facility-based settings. In states like Assam, Uttar Pradesh, Bihar, Gujarat, and Madhya Pradesh, the survey reveals that about half of the pregnancies, around 43–55% were unintended, with the highest proportion of unintended pregnancies in Uttar Pradesh [7].

Unintended pregnancy among adolescents and young women poses significant public health risks, including severe social and economic impacts on the women and their families [8]. Analyzing the situation of unintended pregnancy can be crucial in determining the need for and impact of family planning programs in the country [9]. The important socioeconomic factors influencing unintended pregnancies include age, religion, wealth index, place of residence, number of children, availability and accessibility of contraceptives, and poor knowledge of contraceptive use [10, 11]. Concerning the availability and accessibility of contraceptives, the national and state governments have employed numerous prevention strategies such as health education, skills-building, and improving accessibility to contraceptives worldwide to address this problem [12]. Many kinds of research have been conducted in Africa, Latin America and other developing countries, keeping in mind the importance of sexual and reproductive behavior and the rights of adolescents and youth in their countries. There are a few studies in India on unintended pregnancies, but none of them have special concerns about the adolescent and young female population [13, 14]. The current study, therefore, aims to study the prevalence of unintended pregnancy, examine the association between sociodemographic variables and unintended pregnancy and analyze the important predictors of these pregnancies among adolescents and young adult females in Uttar Pradesh and Bihar.

## Material and Methods

### Data source and study population

The data used in the present study is taken from the Population Council’s longitudinal survey called 'Understanding the Lives of adolescents and Young Adults (UDAYA) conducted in 2015–16 and 2018–19. UDAYA is a one-of-a-kind cross-sectional and longitudinal state representative survey that gathered information on various socioeconomic, demographic, and personal factors and shed light on factors determining transitions to adulthood [15]. UDAYA is based on both cross-sectional and longitudinal designs. The cross-sectional component entails interviewing independent samples of unmarried boys in ages 10–14 and 15–19, unmarried girls in ages 10–14 and 15–19 and married girls in ages 15–19 in both rural and urban settings in Bihar and Uttar Pradesh at two points in time. The first round of cross-sectional surveys was conducted in 2015–16 and the second round was conducted in 2018–19. The longitudinal component comprises: (1) re-interviewing in 2015–16 the sample of unmarried girls and boys and married girls in ages 15–19 who were first interviewed in 2007 as part of the Youth Study and were at the time of the re-interview in ages 23–27 in Bihar; and (2) re-interviewing in 2018–19 the sample of adolescents who were interviewed for the first time in 2015–16 in Bihar and Uttar Pradesh. A total of 10,433 adolescents in Bihar and 10,141 adolescents in Uttar Pradesh were interviewed in 2015–16, and a follow-up interview in 2018–19 was executed [16].

Unintended pregnancy among adolescent girls and adult females was the outcome variable of the study. The question on the intendedness of the pregnancy was asked to only currently married adolescents who were currently pregnant at the time of the survey. They were asked the question, “You are currently pregnant; did you want this pregnancy now, later or not at all?

The response included three options (a) pregnancy wanted at the time of conception (wanted), (b) pregnancy wanted later (mistimed) and (c) pregnancy not wanted at all (unwanted). These three categories were refined and divided into two categories: intended (wanted) and unintended pregnancies (mistimed and unwanted). The outcome variable was coded as 0 = “Intended pregnancy” and 1 = “Unintended pregnancy”.

Currently pregnant adolescents and young adults of age 16–23 were included in the study. The study’s final sample included 659 (286 at Wave 1 and 373 at Wave 2) adolescents and young adults in Uttar Pradesh and 1321 (586 at Wave 1 and 735 at Wave 2) adolescents and young adults in Bihar who were currently pregnant at the time of the survey. The study is based on a secondary dataset; hence, no ethical approval from any institutional board was required. The Institutional Review Board approved the study and its data collection by the Population Council. It also ensured the confidentiality of the participants and informed consent was sought from the respondents during the survey.

### Predictors

In order to determine the factors affecting unintended pregnancies among adolescents in Bihar and Uttar Pradesh, socioeconomic, demographic, health and contraception-related variables were taken into the analysis. The socioeconomic and demographic factors included caste (categorized as SC/ST & Others), wealth index (categorized as Rich, Middle & Poor), place of residence (categorized as Urban & Rural), religion (categorized as Hindu & Non-Hindu), education of the respondent (categorized as illiterate &, literate), age of the respondent (categorized as 16–19 & 20–23), age of the spouse (categorized as <  = 18, 19 to 25, 26 and more & don’t know), husband’s year of schooling (categorized as no schooling, 1–7 years, 8–9 years & 10 years and above), media use (categorized as No & Yes) and internet use (categorized as No & Yes). The demographic and contraception-related factors include ever given birth to a live child (categorized as no, yes & first time pregnant), number of wanted children (categorized as, less than 2 & more than 2), knowledge and use of contraception (categorized as no & yes), side effects of contraception (categorized as no & yes) allowed to go to a health facility (categorized as No & Yes), can obtain information about contraceptives from ASHA/ANM (categorized as confidant & not confidant), knowledge about termination of pregnancy (categorized as No & Yes), discussion about number of children (categorized as No & Yes), heard about SATHIYA (social workers focusing on improving the lives of girls across local communities by providing them education on their health related issues) (categorized as No & Yes), discussion about the number of children (categorized as no & yes) and heard about Adolescent Friendly Health Clinic (categorized as No & Yes).

### Statistical analysis

The study starts with the univariate analysis (sample distribution) for all the variables taken in the analysis for Wave 1 and Wave 2 in Uttar Pradesh and Bihar. Afterwards, a bivariate analysis was conducted to examine the association between the dependent and the independent variables. A Chi-square test tested the degree of association for the same. Finally, a binary logistic regression model was used to analyze the adjusted effect of the predictors on unintended pregnancy among adolescent and young adult females represented as,

$$\mathrm{Logit}\;(\mathrm p)=ln\frac{\mathrm p}{1-\mathrm p}b_0+b_1x_1+b_2x_2+b_3x_3\dots\dots+b_ix_i$$

where ${X}_{i}$ are predictor variables and ${b}_{1}, {b}_{2}, {b}_{3}\dots \dots {b}_{i}$ represents the coefficient of each predictor variable included in the model. The logistic regression was interpreted in terms of odds ratio, which showed how likely or unlikely the outcome was to be present among those with X = 0 and those with X = 1. The statistical software used for the entire process of data analysis was STATA (Version 15).

## Results

### Sample characteristics

Table 1 presents the description of the profile of the respondents in Uttar Pradesh at Wave 1 and Wave 2. At both Wave 1 and Wave 2, most females belonged to the age group 16–19 (99.7% & 95.9%). Caste-wise, most of the respondents belonged to Other caste, followed by the Scheduled caste at both Wave 1 and Wave 2. Most adolescents belonged to the Hindu religion at both periods, while the number of literate adolescents decreased from Wave 1 to Wave 2. More than half of the adolescents at Wave 1 belonged to the poor wealth index, whereas 51.9 per cent of the adolescents belonged to the rich wealth index at Wave 2. Most respondents resided in the rural areas at Wave 1 and Wave 2. At Wave 1, 79.2 per cent of adolescents were exposed to media, which increased to 89.2 per cent at the time of Wave 2. Internet use among adolescents drastically increased from 0.9 per cent at Wave 1 to 24.8 per cent at Wave 2. The majority of the adolescents were first-time pregnant at Wave 1. At the same time, 60.4 per cent had ever given birth to a live child at the time of Wave 2. 64.4 per cent and 67.7 per cent of adolescents preferred less than 2 children at Wave 1 and Wave 2, respectively. Most adolescents were not allowed to go to a health facility at both Wave 1 and Wave 2. Most adolescents have heard about contraception at both periods, while 98.4% did not hear about SATHIYA at Wave 2. Almost 91 per cent of the females had ever used any method of contraception at Wave 1, which decreased to 36.3 at Wave 2. Most adolescents felt confident about obtaining information about contraceptives from ASHA/ANMs at Wave 1 and Wave 2. More than half (51.5%) of the adolescents at Wave 1 thought contraceptive methods would cause side effects, while 53.3 per cent at Wave 2 said no to this notion. Most respondents at both periods believed that a woman could not terminate the pregnancy. At Wave 1, almost 57 per cent of the respondents discussed the number of children with their husbands, which decreased to only 17.4 per cent at Wave 2. Lastly, most adolescents did not hear about the friendly health clinics at Wave 1 (94.2%) and Wave 2 (95.6%).

**Table 1:** Percentage distribution of background characteristics of sample population of Uttar Pradesh, UDAYA, 2015–19

**Predictors**	**Uttar Pradesh**			
	**Wave 1 (2015–16)**		**Wave 2 (2018–19)**	
	**Frequency**	**Per cent**	**Frequency**	**Per cent**
**Age**				
16–19	285	99.7	358	95.9
20–23	1	0.3	15	4.1
**Caste**				
SC/ST	90	31.6	128	34.3
Other	196	68.4	245	65.7
**Religion**				
Hindu	211	73.9	286	76.6
Non-Hindu	75	26.2	87	23.4
**Education**				
Illiterate	3	1.1	8	2.3
Literate	283	98.9	365	97.7
**Wealth Index**				
Rich	82	28.5	194	51.9
Middle	50	17.5	74	19.9
Poor	154	53.9	105	28.2
**Residence**				
Urban	48	16.8	40	10.8
Rural	238	83.2	333	89.2
**Media**				
No	60	20.8	65	17.5
Yes	226	79.2	308	82.5
**Internet use**				
No	283	99.1	92	24.8
Yes	3	0.9	281	75.2
**Ever given birth to a live child**				
No	101	35.2	73	19.5
Yes	58	20.1	225	60.4
First time pregnant	128	44.7	75	20.1
**Number of wanted children**				
Less than 2	184	64.4	252	67.7
More than 2	102	35.6	121	32.4
**Allowed to go to a health facility**				
No	205	71.6	286	76.8
Yes	81	28.4	87	23.3
**Heard about contraception**				
No	11	3.9	55	14.8
Yes	275	96.1	314	85.2
**Heard about SATHIYA**				
No	NA	NA	367	98.4
Yes	NA	NA	6	1.7
**Ever used any method**				
No	25	8.8	238	63.7
Yes	261	91.2	135	36.3
**Can obtain info about contraceptives from ASHA/ ANM**				
Confidant	255	89.2	327	87.7
Not confidant	31	10.8	46	12.3
**Thought contraceptive method would have side effects**				
No	138	48.5	197	53.3
Yes	146	51.5	172	46.8
**A pregnant woman/girl can terminate her pregnancy**				
No	200	69.9	327	87.7
Yes	86	30.1	46	12.4
**Husband wife discussed about number of children**				
No	123	43.1	308	82.6
Yes	163	56.9	65	17.4
**Husband’s number of schooling**				
No schooling	47	16.4	19	12.1
1 to 7 years	51	17.8	12	7.6
8 to 9 years	96	33.7	39	24.0
10 years and above	92	32.2	91	56.3
**Age of spouse**				
< = 18	9	3.0	11	2.9
19 to 25	237	82.9	91	24.4
26 and more	24	8.5	22	5.8
Don’t Know	16	5.6	250	66.9
**Heard about Adolescent friendly health clinics**				
No	269	94.2	357	95.6
Yes	17	5.8	16	4.4
**Total**	**286**	**32.8**	**373**	**33.6**

*UDAYA* Understanding the lives of adolescents and young adults, *SC/ST* Scheduled Caste/ Scheduled Tribe, *ASHA* Accredited Social Health Activist, *ANM* Auxiliary Nursing Midwife, *NA* Not Available

Table 2 presents the description of the profile of the respondents in Bihar at Wave 1 and Wave 2. At Wave 1, the majority of the respondents (46.6%) were 20–23-year-old while at Wave 2 (84.8%) majority were 16–19-year-old. Most of the respondents belonged to Other caste and Hindu religion and were literate at both Wave 1 and Wave 2. At Wave 1, most respondents hailed from the rich wealth quantile (44.4%), whereas the majority belonged to the poor wealth quantile (48.1%) at Wave 2. Most of the respondents were rural residents and were exposed to media during both periods. At Wave 1, 39.8 per cent of the adolescents were first-time pregnant, while 62.2 per cent had ever given birth to a live child at the time of Wave 2. More than 60 per cent of adolescents preferred less than 2 children at both Wave 1 and Wave 2.

**Table 2:** Percentage distribution of background characteristics of sample population of Bihar, UDAYA, 2015–19

**Predictors**	**Bihar**			
	**Wave 1 (2015–16)**		**Wave 2 (2018–19)**	
	**Frequency**	**Percent**	**Frequency**	**Percent**
**Age**				
16–19	273	46.6	623	84.8
20–23	313	53.4	112	15.2
**Caste**				
SC/ST	158	26.9	203	27.6
Other	428	73.1	532	72.4
**Religion**				
Hindu	525	89.6	642	87.3
Non-Hindu	61	10.3	93	12.7
**Education**				
Illiterate	10	1.7	15	2.0
Literate	576	98.3	720	98.0
**Wealth Index**				
Rich	262	44.7	194	26.3
Middle	140	23.9	188	25.5
Poor	184	31.5	354	48.1
**Residence**				
Urban	66	11.2	67	9.1
Rural	520	88.8	668	91.0
**Media**				
No	193	32.9	207	28.1
Yes	393	67.1	528	71.9
**Internet use**				
No	578	98.7	287	39.0
Yes	8	1.3	448	61.0
**Ever given birth to a live child**				
No	141	24.1	108	14.7
Yes	212	36.1	457	62.2
First time pregnant	233	39.8	170	23.1
**Number of wanted children**				
Less than 2	363	61.9	482	65.6
More than 2	223	38.1	253	34.4
**Allowed to go to a health facility**				
No	404	69.0	585	79.6
Yes	182	31.1	150	20.4
**Heard about contraception**				
No	58	9.9	167	23.0
Yes	528	90.1	557	77.0
**Heard about SATHIYA**				
No	NA	NA	726	98.8
Yes	NA	NA	9	1.2
**Ever used any method**				
No	47	8.0	598	81.4
Yes	539	92.0	137	18.6
**Can obtain info about contraceptives from ASHA/ ANM**				
Confidant	505	86.2	645	87.8
Not confidant	81	13.8	90	12.2
**Thought contraceptive method would have side effects**				
No	216	37.0	292	40.0
Yes	369	63.0	439	60.0
**Pregnant woman/girl can terminate pregnancy**				
No	358	61.1	596	81.1
Yes	228	38.9	139	18.9
**Husband wife discussed about number of children**				
No	265	45.3	563	76.6
Yes	321	54.7	172	23.4
**Husband’s number of schooling**				
No schooling	150	25.6	42	17.2
1 to 7 years	117	20.0	39	15.8
8 to 9 years	130	22.2	52	21.3
10 years and above	189	32.3	111	45.7
**Age of spouse**				
< = 18	14	2.4	24	3.3
19 to 25	437	74.5	175	23.8
26 and more	64	11	39	5.3
Don’t Know	71	12.1	497	67.6
**Heard about Adolescent friendly health clinics**				
No	550	93.8	693	94.4
Yes	36	6.2	42	5.7
**Total**	**586**	**67.2**	**735**	**66.3**

*UDAYA* Understanding the lives of adolescents and young adults, *SC/ST* Scheduled Caste/ Scheduled Tribe, *ASHA* Accredited Social Health Activist, *ANM* Auxiliary Nursing Midwife, *NA* Not Available

Many respondents were not allowed to attend a health facility at either period. Most adolescents have heard about contraception at both periods. In contrast, only 1.2% had heard about SATHIYA at Wave 2. 92 per cent of the respondents had ever used any method of contraception at Wave 1, while it was only 18.6 per cent at Wave 2. Most of the adolescents felt confident about obtaining information about contraceptives from ASHA/ANMs at both periods. More than 60 per cent of the adolescents at both periods responded that contraceptive methods would cause side effects. Most respondents at both periods believed that a woman could not terminate the pregnancy. At Wave 1, almost 55 per cent of the respondents discussed the number of children with their husbands, which decreased to only 24 per cent at Wave 2. Most respondents (74.5%) reported their spouse’s age as 19–25, whereas the majority did not know about their spouse’s age at Wave 2 (67.6%). Lastly, more than 93 per cent of the adolescents did not hear about the adolescent-friendly health clinics at both waves.

**Fig. 1:**
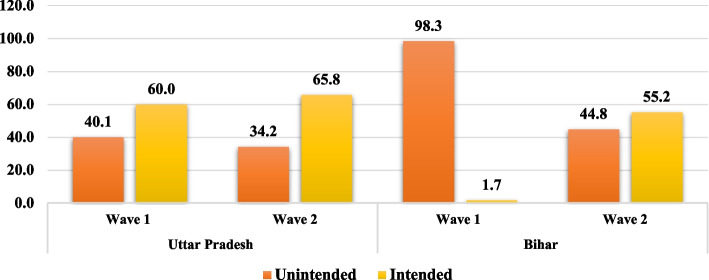
Prevalence of Unintended Pregnacy among adolescents age 16–23 in Uttar Pradesh and Bihar, UDAYA, 2015–2019

Figure 1 shows that 40.1 per cent of all currently pregnant adolescents and young adults reported their pregnancy as unintended (mistimed and unwanted) in Uttar Pradesh at Wave 1 of the survey, which decreased to 34.2 per cent at Wave 2. On the contrary, almost 99 per cent of all currently pregnant adolescents and young adults in Bihar reported their pregnancy as unintended at Wave 1, which decreased to 44.8 per cent at Wave 2.

### Association of unintended pregnancy and predictors among adolescents in Uttar Pradesh at Wave 1 and 2

Table 3 presents the bivariate analysis in which age and unintended pregnancy were positively associated among adolescents, wherein the prevalence of unintended pregnancy was highest among the 20–23 age group in Uttar Pradesh at Wave 2. Religion-wise, the highest prevalence of unintended pregnancy was observed among Non-Hindu adolescents at both Wave 1 (48.0%) and Wave 2 (38.2%). It was also observed that the prevalence of unintended pregnancy was higher among illiterate adolescents at Wave 1 (78.5%). Wealth and unintended pregnancies were positively related as an increase in unintended pregnancies was observed with an increase in the wealth quantile among the adolescents of Uttar Pradesh at Wave 1 (44.8%). At Wave 2, a significant rural–urban difference was observed as adolescents from urban areas showed a significant preponderance of unintended pregnancy (38.6%). Exposure to media and internet use was significantly associated with a lower prevalence of unintended pregnancy among adolescents at both periods. At Wave 1, a higher prevalence of unintended pregnancy was found among first-time pregnant adolescents (41%). At both Wave 1 and 2, unintended pregnancy was higher among adolescents who preferred less than two children. Adolescents who were not allowed to go to a health facility reported higher unintended pregnancies at both periods. At Wave 2, adolescents who had not heard about contraception reported higher unintended pregnancy (34.3%). Subsequently, the prevalence of unintended pregnancy was also higher among the respondents who did not use any method of contraception (55%) at Wave 1. Unintended pregnancy was observed high among females who thought contraceptive methods would have side effects (36.6%) at Wave 2. The prevalence of unintended pregnancy was higher at both waves among women who thought a pregnant woman could not terminate a pregnancy. At both Wave 1 and Wave 2, a higher prevalence of unintended pregnancy was found when the husband and wife did not discuss about the number of children (48.7% & 35.1%). A positive relationship was found between the husband’s education and age with unintended pregnancy at Wave 2. Lastly, a higher prevalence of unintended pregnancy was observed among adolescents who had not heard about adolescent-friendly health clinics at both periods (41.1% & 35).

**Table 3:** Socioeconomic and demographic factors associated with unintended pregnancy among adolescents of Uttar Pradesh, UDAYA, 2015–19

**Predictors**	**Uttar Pradesh**			
	**Wave 1 (2015–16)**		**Wave 2 (2018–19)**	
	**Unintended pregnancy**	***P-value***	**Unintended pregnancy**	***P-value***
**Age**				(< 0.001)
16–19	39.9		33.9	
20–23	100		41.97	
**Caste**				
SC/ST	33.7		32.4	
Other	43.0		35.2	
**Religion**		(< 0.001)		(< 0.001)
Hindu	37.2		33.0	
Non-Hindu	48.0		38.2	
**Education**		(< 0.001)		
Illiterate	78.5		15.2	
Literate	39.6		34.7	
**Wealth Index**		(< 0.001)		(< 0.001)
Rich	29.4		34.4	
Middle	42.7		38.6	
Poor	44.8		30.9	
**Residence**				(< 0.001)
Urban	39.4		35.8	
Rural	40.2		34.0	
**Media**		(< 0.001)		(< 0.001)
No	41.1		35.7	
Yes	36.2		33.9	
**Internet Use**		(< 0.001)		(< 0.001)
No	75.0		35.9	
Yes	39.7		29.1	
**Ever given birth to a live child**		(< 0.001)		
No	38.7		19.9	
Yes	40.3		40.6	
First time pregnant	41		29.0	
**Number of wanted children**		(< 0.001)		(< 0.001)
Less than 2	42.6		36.3	
More than 2	35.5		29.9	
**Allowed to go to a health facility**		(< 0.001)		
No	49.6		34.6	(< 0.001)
Yes	36.3		33.1	
**Heard about contraception**				
No	31.3		34.3	(< 0.001)
Yes	40.4		32.8	
**Heard about SATHIYA**				
No	NA		33.9	(< 0.001)
Yes	NA		51.6	
**Ever used any method**		(< 0.001)		
No	55.0		35.7	
Yes	38.6		31.7	
**Can obtain info about contraceptives from ASHA/ ANM**		(< 0.001)		
Confidant	36.0		35.4	(< 0.001)
Not confidant	40.5		25.7	
**Thought contraceptive method would have side effects**		(< 0.001)		
No	40.7		32.7	
Yes	39.8		36.6	
**Pregnant woman/girl can terminate pregnancy**		(< 0.001)		
No	40.4		48.1	(< 0.001)
Yes	39.2		32.3	
**Husband wife discussed about number of children**		(< 0.001)		
No	48.7		35.1	(< 0.001)
Yes	28.6		30.5	
**Husband’s number of schooling**				
No schooling	32.6		71.2	(< 0.001)
1 to 7 years	41.9		63.9	
8 to 9 years	46.1		69.3	
10 years and above	36.5		57.2	(< 0.001)
**Age of spouse**				
< = 18	35.9		20.1	
19 to 25	38.9		34.8	
26 and more	70.1		35.2	
Don’t Know	14.6		34.6	(< 0.001)
**Heard about Adolescent friendly health clinics**		(< 0.001)		
No	41.1		35.0	
Yes	22.9		17.7	

*UDAYA* Understanding the lives of adolescents and young adults, *SC/ST* Scheduled Caste/ Scheduled Tribe, *ASHA* Accredited Social Health Activist, *ANM* Auxiliary Nursing Midwife, *NA* Not Available

*P*-values are based on Chi-square

### Association of unintended pregnancy and predictors among adolescents in Bihar at Wave 1 and 2

Table 4 presents the bivariate analysis results starting with age significantly associated with unintended pregnancy in Bihar at the time of both Wave 1 and Wave 2. A higher prevalence of unintended pregnancy was found among non-Hindu adolescents at Wave 1 (53.5%) and among Hindu adolescents at Wave 2 (46%). Adolescents who were literate and those who resided in urban areas showed a higher preponderance of unintended pregnancy at Wave 1 and Wave 2, respectively. Wealth index was positively associated with unintended pregnancy as unintended pregnancy showed a decrement among adolescents with a decrease in wealth quantiles. Media exposure and internet use were significantly associated with a lower prevalence of unintended pregnancy among adolescents at both periods (46.1% & 43.5%). At both Wave 1 and 2 a higher prevalence of unintended pregnancy was found among adolescents who had given birth to a live child. Adolescents who preferred less than two children showed a higher preponderance of unintended pregnancy at both waves. A higher prevalence of unintended pregnancy was observed among adolescents who were not allowed to go to a health facility at both periods (48.6% & 45.4%). A higher preponderance of unintended pregnancy was found among adolescents who have not heard about contraception at Wave 2 (45.5%). Moreover, the prevalence of unintended pregnancy was also higher among the respondents who did not use any method of contraception at both periods. At Wave 1, adolescents confident in obtaining information about contraceptives from ASHA/ANM showed less preponderance of unintended pregnancy (45.8%). The prevalence of unintended pregnancy was higher among those who thought contraceptive methods would have side effects (36.6%) at both Wave 1 and Wave 2. Similarly, the prevalence of unintended pregnancy was higher at both waves among women who thought pregnant women could not terminate a pregnancy. Unintended pregnancy was higher among respondents who did not discuss the number of children with their spouses during both periods. Lastly, a higher prevalence of unintended pregnancy was observed among adolescents who had not heard about adolescent-friendly health clinics during both periods (47% & 45).

**Table 4:** Socioeconomic and demographic factors associated with unintended pregnancy among adolescents of Bihar, UDAYA, 2015–19

**Predictors**	**Bihar**			
	**Wave 1 (2015–16)**		**Wave 2 (2018–19)**	
	**Unintended Pregnancy**	***P-value***	**Unintended Pregnancy**	***P-value***
**Age**		(< 0.001)		(< 0.001)
16–19	46.7		42.0	
20–23	42.8		60.3	
**Caste**				
SC/ST	46.4		41.0	
Other	46.7		46.3	
**Religion**		(< 0.001)		(< 0.001)
Hindu	45.8		46.0	
Non-Hindu	53.5		36.8	
**Education**		(< 0.001)		
Illiterate	29.6		23.3	
Literate	46.9		45.3	
**Wealth Index**		(< 0.001)		(< 0.001)
Rich	46.8		53.2	
Middle	51.4		44.3	
Poor	42.6		40.5	
**Residence**				(< 0.001)
Urban	52.2		51.2	
Rural	45.9		44.2	
**Media**		(< 0.001)		(< 0.001)
No	47.0		37.4	
Yes	45.9		47.7	
**Internet Use**		(< 0.001)		(< 0.001)
No	83.9		46.8	
Yes	46.1		43.5	
**Ever given birth to a live child**		(< 0.001)		(< 0.001)
No	47.9		29.4	
Yes	53.5		50.5	
First time pregnant	39.5		39.3	
**Number of wanted children**		(< 0.001)		(< 0.001)
Less than 2	48.3		48.1	
More than 2	43.9		38.6	
**Allowed to go to a health facility**		(< 0.001)		(< 0.001)
No	48.6		45.4	
Yes	42.2		44.6	
**Heard about contraception**				(< 0.001)
No	51.5		45.5	
Yes	46.1		39.5	
**Heard about SATHIYA**				
No	NA		44.7	
Yes	NA		49.9	
**Ever used any method**		(< 0.001)		(< 0.001)
No	67.5		48.0	
Yes	44.8		44.1	
**Can obtain info about contraceptives from ASHA/ ANM**		(< 0.001)		
Confidant	45.8		45.6	
Not confidant	51.8		39.1	
**Thought contraceptive method would have side effects**		(< 0.001)		(< 0.001)
No	47.7		43.3	
Yes	45.8		47.1	
**Pregnant woman/girl can terminate pregnancy**		(< 0.001)		(< 0.001)
No	50.0		46.9	
Yes	41.3		36.0	
**Husband wife discussed about number of children**		(< 0.001)		(< 0.001)
No	54.0		44.8	
Yes	37.7		44.9	
**Husband’s number of schooling**				(< 0.001)
No schooling	47.6		70.4	
1 to 7 years	47.8		53.1	
8 to 9 years	41.1		42.2	
10 years and above	48.8		47.4	
**Age of spouse**				(< 0.001)
< = 18	50.7		39.7	
19 to 25	47.7		54.7	
26 and more	36.4		54.9	
Don’t Know	48.5		40.8	
**Heard about Adolescent friendly health clinics**		(< 0.001)		(< 0.001)
No	47.0		45.0	
Yes	40.0		42.4	

*UDAYA* Understanding the lives of adolescents and young adults, *SC/ST* Scheduled Caste/ Scheduled Tribe, *ASHA* Accredited Social Health Activist, *ANM* Auxiliary Nursing Midwife, *NA* Not Available

*P*-values are based on Chi-square

### Predictors of unintended pregnancies among adolescents in Uttar Pradesh and Bihar

Table 5 presents the results of binary logistic regression analyses showing the relation of pregnancy intention of adolescents and young adults' most recent birth by controlling for socioeconomic and demographic variables. The results indicated that factors like husband’s schooling, age of spouse, allowed to go to a health facility, and termination of pregnancy were not significantly associated with the pregnancy intention status of the most recent birth (mistimed or unwanted) at both Wave 1 and 2, when all covariates were adjusted.

**Table 5:** Binary Logistic regression analyses showing the predictors of unintended pregnancy among adolescents of Bihar and Uttar Pradesh, UDAYA, 2015–19

**Predictors**	**Wave 1 (2015–16)**		**Wave 2 (2018–19)**	
	**OR [95% CI]**	***P-value***	**OR [95% CI]**	***P-value***
**Age**				
16–19 **®**	1		1	
20–23	1.31 [1.15–1.50]	0.000	0.64 [0.44–0.95]	0.000
**Caste**				
SC/ST**®**	1			
Other	1.58 [1.23–2.05]	0.000	0.95[ 0.73–1.24]	0.000
**Religion**				
Hindu**®**	1			
Non-Hindu	1.58 [1.13–2.09]	0.000	1.27 [0.92–1.75]	0.000
**Education**				
Illiterate**®**	1			
Literate	1.36 [0.48–3.35]	0.000	0.42 [0.15–1.16]	0.000
**Wealth Index**				
Rich**®**	1		1	
Middle	0.72 [0.50–1.04]	0.076	0.79[ 0.58–1.09]	0.156
Poor	1.54 [1.23–1.93]	0.000	1.65 [1.35–2.02]	0.000
**Residence**				
Urban**®**	1		1	
Rural	1.19 [0.91–1.57]	0.212	1.19 [0.93–1.53]	0.000
**Media**				
No**®**	1		1	
Yes	0.84	0.000	0.740 [0.55–0.99]	0.000
**Internet Use**				
No**®**	1		1	
Yes	0.21 [0.07–0.71]	0.011	0.975 [0.76–1.25]	0.000
**Ever given birth to a live child**				
No**®**	1		1	
Yes	0.51 [0.35–0.75]	0.000	0.37 [0.26–0.55]	0.000
First time pregnant	1.05 [0.74–1.50]	0.777	0.77 [0.50–1.20]	0.246
**Number of wanted children**				
Less than 2**®**	1		1	
More than 2	1.31 [1.00–1.73]	0.054	1.05 [0.82–1.35]	0.000
**Allowed to go to a health facility**				
No**®**	1		1	
Yes	0.94 [0.71–1.26]	0.709	1.06 [0.81–1.41]	0.648
**Heard about contraception**				
No**®**	1		1	
Yes	0.87 [0.53–1.45]	0.615	0.89 [0.65–1.22]	0.000
**Heard about SATHIYA**				
No**®**	NA	NA	1	
Yes	NA	NA	0.45 [0.18–1.19]	0.000
**Ever used any method**				
No**®**	1		1	
Yes	1.84 [1.14–2.98]	0.012	0.98 [0.74–1.30]	0.000
**Can obtain info about contraceptives from ASHA/ ANM**				
Confidant**®**	1		1	
Not confidant	1.33 [1.15–1.54]		1.44 [1.01–2.06]	0.000
**Thought contraceptive method would have side effects**				
No**®**	1		1	
Yes	1.31 [1.07–1.62]	0.008	1.36 [1.15–1.62]	0.000
**Pregnant woman/girl can terminate pregnancy**				
No**®**	1		1	
Yes	1.06 [0.80–1.41]	0.668	0.81 [0.60 = 1.10]	0.185
**Husband wife discussed about number of children**				
No**®**	1		1	
Yes	0.50 [0.38–0.69]	0.000	1.33 [1.17–1.52]	0.000
**Husband’s number of schooling**				
No schooling**®**	1		1	
1 to 7 years	1.35 [1.04–1.78]	0.026	1.00 [0.48–2.13]	0.986
8 to 9 years	1.05 [0.72–1.56]	0.773	1.21 [0.63–2.37]	0.560
10 years and above	0.81 [0.57–1.17]	0.275	0.89 [0.50–1.60]	0.698
**Age of spouse**				
< = 18 **®**	1		1	
19 to 25	1.03 [0.46–2.34]	0.933	0.51 [0.22–1.21]	0.127
26 and more	1.51 [0.61–3.75]	0.372	0.46 [0.18–1.20]	0.114
Don’t Know	1.37 [0.54–3.51]	0.501	0.55 [0.24–1.28]	0.171
**Heard about Adolescent friendly health clinics**				
No**®**	1		1	
Yes	0.74 [0.47–1.18]	0.000	1.36 [1.21–1.54]	0.000

*UDAYA* Understanding the lives of adolescents and young adults, *SC/ST* Scheduled Caste/ Scheduled Tribe, *ASHA* Accredited Social Health Activist, *ANM* Auxiliary Nursing Midwife, *NA* Not Available, *OR* Odds Ratio, *CI* Confidence Interval

® Reference category; *p* < .001

Older adolescents were 31 per cent more likely to have higher unintended pregnancies at Wave 1, whereas adolescents aged 20–23 were 36 per cent less likely to have unintended pregnancies than their counterparts at Wave 2. Adolescents of Other caste were more likely to have unintended pregnancy at Wave 1 while they were 15 per cent significantly less likely [OR = 0.95, 95% CI = 0.73–1.24] to have an unintended pregnancy compared to SC\ST caste at Wave 2. Religion-wise, adolescents from non-Hindu religion were 58 per cent and 27 more likely to have unintended pregnancy at both periods. Literate adolescents were 36 per cent more likely than the illiterate ones to have reported their current pregnancy as unintended at Wave 1 [OR = 1.36, 95% CI = 0.48–3.85]. On the other hand, the literate adolescents were 58 per cent less likely than the illiterate ones to have reported their current pregnancy as unintended at Wave 2. Wealth and unintended pregnancy showed a significant negative relationship at both periods. Adolescents from poor wealth quantiles were significantly more likely to have unintended pregnancies than their affluent counterparts at Waves 1 and 2. Adolescents from rural backgrounds were 19 per cent more likely to have an unintended pregnancy than their urban counterparts at Wave 2. In case of media exposure, adolescents exposed to media were 16 per cent and 26 per cent less likely to report their pregnancy as unintended than their counterparts at Wave 1 and 2, respectively. Comparatively, those who used the internet were 3 per cent significantly less likely to report an unintended pregnancy at Wave 2. Adolescents who had ever given birth to a live child were significantly less likely to have unintended pregnancies than those who had not given birth at both periods. Adolescents who wanted more than two children were 5 per cent significantly more likely to have an unintended pregnancy than their counterparts [OR = 1.05, 95% CI = 0.82–1.35]. Adolescents who heard about contraception and SATHIYA were 11 per cent and 55 per cent significantly less likely to have unintended pregnancies at Wave 2, respectively. Adolescents who were not confident about getting contraceptive information from ASHA/ANM and those who thought contraceptive methods would have caused side effects were significantly more likely to have unintended pregnancy at Wave 2. Adolescents who discussed the number of children with their spouses were 50 per cent less likely and 33 per cent more likely to have unintended pregnancy at Wave 1 and 2, respectively. Lastly, adolescents who heard about adolescent-friendly health clinics were 26 per cent less likely and 36 per cent more likely to have unintended pregnancies at Wave 1 and 2, respectively.

## Discussion

Adolescent health and wellbeing constitute vital components of the Sustainable Development Goals (SDGs), aiming to accomplish economic, social and environmentally sustainable development by 2030. It includes planning and programming policies for adolescents’ health and wellbeing, focusing on their special unmet needs and demands. Aligning with the Sustainable Development Goals, the government of India launched the Global Strategy for Women’s, Children’s and Adolescents’ Health (2016–2030) in 2015. It provides a unique platform for improving adolescent health and responding more effectively to adolescents’ unique needs [17]. The present study highlights an important issue of unintended pregnancy among adolescent and young adult females from two less empowered states of India. The study estimated the levels of unintended pregnancy among adolescents about socioeconomic, demographic, contraception and health-related factors, using the longitudinal data of the UDAYA survey in Bihar and Uttar Pradesh. The study revealed the association between socioeconomic, demographic and other factors with unintended pregnancies among adolescents and young adults [18]. Further, it showed the crucial predictors affecting unintended pregnancy among adolescents in Bihar and Uttar Pradesh from 2015 to 2019.

The results revealed that around 41 and 35 per cent of pregnant adolescent and young adults in Uttar Pradesh report their current pregnancy as unintended at Wave 1 and 2, respectively. It is surprising to notice that approximately 99 per cent of all the current pregnancies in Bihar were reported as unintended. This was due to highly skewed sample size at Wave 1. However, this scenario changed at Wave 2, where around 45 per cent of the pregnancies were reported as unintended. Another plausible reason for this decline can be attributed to the awareness and information imparted by the health care workers like ASHA/ANMs in the private as well as the community settings [19, 20]. Unintended pregnancy leads to several negative consequences of maternal and child health complications and puts unnecessary pressure on the government in the form of financial expenses [21]. In this scenario, such a high percentage of adolescents in Bihar and Uttar Pradesh reporting their current pregnancy as unintended embodies a significant gap between the expected and met needs of adolescents and young adult females.

Age constituted an important element amongst the influential socioeconomic factors affecting unintended pregnancy. Both young and older age seemed to be affected by unintended pregnancy during the different survey periods in Uttar Pradesh and Bihar. Adolescents 20–23 were more vulnerable and reported their current pregnancy as unintended at Wave 1 [22]. A study conducted in Nepal in 2009 also discovered that higher a woman’s age, the higher the probability of having an unintended pregnancy [23]. In addition to this, adolescents of age 16–19 were more susceptible to unintended pregnancy at Wave 2. Earlier studies have also reported that women from a younger age tend to report their pregnancy as unintended compared to older ones [24, 25].

Caste-based differences were observed in which unintended pregnancy was more often seen among adolescents from Other caste [26]. In this study, non-Hindu religion was significantly associated with the incidence of unintended pregnancy in Uttar Pradesh and Bihar. A plausible explanation could be that non-Hindu women are more likely to accept their pregnancy as “a gift of God” or a “treasure of the family” [23]. Surprisingly, unintended pregnancy was higher among literate adolescents in Uttar and Bihar, while in most studies, education was seen as a predictor of unintended pregnancy wherein unintended pregnancy was observed less among literate women [27]. Wealth index was positively associated with unintended pregnancy among adolescents in Bihar and Uttar Pradesh. Our finding goes in tune with previous studies, which also revealed that unintended pregnancies are seen more among adolescents from high/middle wealth quantile [28]. Being from urban areas correlated with a greater likelihood of reporting a pregnancy as unintended, as reported in earlier studies as well [29, 30].

The present study revealed that media and internet use were significantly associated with a lower prevalence of unintended pregnancy in adolescents in Uttar Pradesh and Bihar. It is pretty clear from past studies that media plays a vital role in reducing unintended pregnancy by providing a more comprehensive range of knowledge and increasing awareness about family planning among the young population [23]. This finding also aligns with few related factors which are awareness about contraception,SATHIYA and adolescent friendly health clinics. The findings revealed that adolescents were more prone to unintended pregnancy when they had not heard about contraception, SATHIYA and Adolescent friendly health clinics. These three findings reveal the importance of awareness and exposure to contraception and family planning related matters among the adolescents and young adult female population in the states of UP and Bihar. Earlier studies have revealed that the higher the level of knowledge and awareness among adolescents about contraception, the fewer the chances of unintended pregnancy [27].

Contraceptive use emerged as one of the most crucial factors in defining unintended pregnancy. Previous studies have observed that lack of contraceptive use is a crucial factor in unintended pregnancy. Many unintended pregnancies occur because effective contraception is not used [31, 32]. A similar finding was observed in the present study as well. Adolescents who have never used any contraception reported more instances of unintended pregnancies. Not using contraception can be attributed to adolescents’ lack of information and knowledge.

The present study also revealed that unintended pregnancy was high among females who thought contraceptive methods would have side effects. The primary reason behind this finding is the widespread myths about perceived side effects and health concerns of contraception as well [33]. Lastly, unintended pregnancy was found lower among adolescents who discussed the desired number of children with their husbands than their respective counterparts. Past research also supports this finding that a couple’s agreement on contraception methods and the number of desired children reduces the possibility of unintended pregnancy [27].

### Strengths and limitations of the study

The strength of the study lies in the fact that it uniquely focuses on the drivers of unintended pregnancy among the young population in those two states which lag behind on the indices of reproductive health and family planning matters. Another strength could be attributed to the fact that it derives data from a survey which is dedicated completely towards understanding the health concerns of this young and vulnerable population in Bihar and Uttar Pradesh. Despite these strengths, there are a few limitations as well. Findings should not be extrapolated to the entire population of the country because the data is not representative of all of India and is restricted to just two socioeconomically backward states. Additionally, because our study is quantitative in nature, we cannot capture the unique social and cultural perspectives on the intendedness of pregnancy among young people that might be acquired from a qualitative investigation.

## Conclusion and policy recommendations

We believe that the present study is unique as it examines the association between unintended pregnancy and sociodemographic factors among adolescent and young adult females in two less empowered states of India from 2015 to 2019. The study’s strength lies in the fact that it goes beyond analyzing the levels of unintended pregnancy among women and includes several sociodemographic factors that explain the intention of current pregnancy among young female population in Bihar and Uttar Pradesh over two waves of the UDAYA survey. India is considered a youth nation, so prioritizing adolescents’ health and wellbeing remains a big concern. Despite many recently launched policies for adolescents and the youth population, this study comprehended that the level of unintended pregnancies in Bihar and Uttar Pradesh stands worrisome. Moreover, there is an added advantage to using longitudinal data, which shows the impact of predictors over time. For instance, variables like place of residence, internet use, number of wanted children, heard about contraception and SATHIYA, use of contraception, side effects of contraception, and the confidence of getting contraceptives from ASHA/ANM did not appear significant predictors at Wave 1. However, they emerge significant over time (Wave 2). On the other hand, factors like husband’s schooling, age of spouse, allowed to go to a health facility and termination of pregnancy were not significantly associated with unintended pregnancy at both the waves. The study revealed that age, caste, religion, education, wealth, media and internet use, knowledge and effective contraception use broadly impact unintended pregnancy in the two socially and economically less empowered states of Bihar and Uttar Pradesh. Therefore, a need for more comprehensive family planning services for adolescents to improve their awareness and knowledge about family planning persists. Unintended pregnancies can be avoided by giving young females more say in the family planning decisions and improving their access to contraceptives. In order to decrease unintended pregnancy and its effects in the future, community education on family planning services for all adolescent and young adult females is required. The focus of policymakers’ strategies should be on raising knowledge of family planning services in Bihar and Uttar Pradesh and empowering adolescents’ autonomy over family planning use.

## Data Availability

The datasets supporting the conclusions of this article are available in the data repository at Harvard data verse, following the link https://dataverse.harvard.edu/dataset.xhtml?persistentId=doi:10.7910/DVN/RRXQNT

## Abbreviations

UPAI: Unintended Pregnancy and Abortion in India
UDAYA: Understanding the lives of adolescents and young adults
ASHA: Accredited Social Health Activist
ANM: Auxiliary Nursing Midwife
SC: Scheduled Caste
ST: Scheduled Tribe
SDG: Sustainable Development GoalsDeclarations
